# Carbamazepine improves perioperative outcomes in patients with tentorial meningiomas

**DOI:** 10.3389/fpain.2026.1762292

**Published:** 2026-04-01

**Authors:** Gaowei Xu, Yuhan Zhu, Jing Zhang, Shandong Jiang, Haitao Peng, Yuan Yuan

**Affiliations:** 1 Department of Nursing, The Second Affiliated Hospital, Zhejiang University School of Medicine, Hangzhou, China; 2 Department of Neurosurgery, The Second Affiliated Hospital, Zhejiang University School of Medicine, Hangzhou, China; 3 Clinical Research Center for Neurological Diseases of Zhejiang Province, Hangzhou, China; 4 Zhejiang University School of Medicine, Hangzhou, China

**Keywords:** analgesics, carbamazepine, neuropathic pain, postoperative pain, tentorial meningiomas

## Abstract

**Background:**

Postoperative pain management is critical for patients undergoing surgery for tentorial meningiomas due to the involvement of trigeminal nerve innervation in this region, which often results in severe pain. This study aimed to evaluate the efficacy and safety of carbamazepine (CBZ) in reducing postoperative pain, analgesic consumption, and hospital stay in these patients.

**Methods:**

This retrospective study included consecutive patients with tentorial meningiomas who underwent surgical resection between March 2021 and August 2023 at the Second Affiliated Hospital of Zhejiang University. Patients were divided into two groups, with the control group receiving nonsteroidal anti-inflammatory drugs (NSAIDs) or opioids as needed for pain management, while the CBZ group was additionally treated with CBZ at a dosage of 200 mg twice daily postoperatively. Data collected included baseline demographics, postoperative inflammatory markers, liver function, length of hospital stay, total hospital charges, analgesic consumption, and daily pain score.

**Results:**

A total of 42 patients were included in the study, with 22 in the control group and 20 in the CBZ group. Although the CBZ group had shorter hospital stays and lower costs, these differences were not statistically significant. The CBZ group reported significantly lower pain scores on postoperative days 2 and 7 (*p* < 0.05). Analgesic consumption was similar in the first 96 h postoperatively (*p* = 0.212), but significantly lower in the CBZ group from 96 h to discharge, particularly for opioids (*p* < 0.05).

**Conclusions:**

CBZ effectively reduces postoperative pain, decreases analgesic use without increasing adverse effects in patients with tentorial meningiomas. These findings support the incorporation of CBZ into multimodal pain management strategies for this patient population.

## Introduction

Tentorial meningiomas are a rare but significant subset of intracranial meningiomas that arise from the dura mater of the tentorium cerebelli, account for approximately 3% to 6% of all intracranial meningiomas and about 30% of posterior fossa meningiomas (1, 2). Although meningiomas are generally benign tumors, their location in the tentorial region can lead to severe neurological symptoms due to their proximity to critical structures such as the brainstem, cranial nerves, and blood vessels (3). Symptoms of tentorial meningiomas often include headache, dizziness, and cranial nerve deficits, all of which can severely impact a patient's quality of life. Surgical resection is the mainstay of treatment for tentorial meningiomas (3). However, due to the complex anatomy of the region, these surgeries are technically challenging and carry a high risk of postoperative complications. One of the most common and debilitating complications is postoperative pain, which can be difficult to manage and can impede the patient's recovery (4). Post-craniotomy pain is defined as “acute headache attributed to craniotomy,” occurring within 7 days following surgery and typically resolving within 3 months (5). Effective pain control is essential to improve postoperative outcomes, enhance rehabilitation, and reduce hospital stay. Despite the availability of standard postoperative analgesics, many patients experience inadequate pain relief, leading to the exploration of alternative pharmacological interventions.

Postoperative pain in neurosurgical patients is a multifactorial issue influenced by the invasiveness of the surgery, the duration of the procedure, and the individual patient's pain threshold (6). Since the sensation of the tentorium cerebelli is innervated by branches of the trigeminal nerve, patients with tentorial meningiomas may experience more severe postoperative pain, similar to trigeminal neuralgia (7). Standard pain management typically involves the use of nonsteroidal anti-inflammatory drugs (NSAIDs) and opioids. However, these medications come with limitations (8). NSAIDs may increase the risk of bleeding, particularly in neurosurgical patients. Opioids, while effective, carry a significant risk of adverse effects such as respiratory depression, sedation, and addiction potential (9). Acetaminophen, while safer, is often insufficient for managing severe postoperative pain. Given these limitations, there is a growing interest in exploring adjuvant therapies that can provide more effective pain relief with fewer side effects.

Anticonvulsants, such as carbamazepine (CBZ), have emerged as potential candidates for managing postoperative pain due to their neuromodulatory properties, which is traditionally used to treat epilepsy and trigeminal neuralgia (10, 11). Compared with other anticonvulsants such as gabapentin or pregabalin, CBZ exerts its analgesic effect mainly through the inhibition of voltage-gated sodium channels, thereby stabilizing hyperexcitable neuronal membranes and reducing repetitive firing (6). This mechanism may be particularly relevant in acute postoperative settings characterized by nerve irritation. Several studies have demonstrated the efficacy of CBZ in managing neuropathic pain conditions such as trigeminal neuralgia, for which CBZ is considered a first-line treatment, a disorder that shares similar pathophysiological mechanisms with the pain experienced after tentorial meningioma surgery (12, 13). While its use in postoperative pain is less well-established, there is growing evidence that CBZ may be beneficial in this setting, particularly when standard analgesics fail to provide adequate relief (13–16).

Given the unique challenges of managing postoperative pain in patients undergoing tentorial meningioma resection, there is a need for more effective, targeted interventions. This study aims to evaluate the efficacy of CBZ in reducing postoperative pain in this patient population. By focusing on patients with tentorial meningiomas, this study addresses a gap in the current literature and explores the potential of CBZ as an adjunct to standard postoperative pain management protocols. The hypothesis is that CBZ, due to its action on sodium channels and its proven efficacy in treating neuropathic pain, will significantly reduce postoperative pain in patients following tentorial meningioma surgery. Furthermore, exploring the use of CBZ in this context could open the door to broader applications of anticonvulsants in neurosurgical postoperative care.

## Methodology

### Study design and study subjects

This is a retrospective analysis of consecutive patients with tentorial meningiomas who underwent surgery from 1st March 2021 to 31stAugust 2023. Patients included in the study were required to have a confirmed diagnosis of tentorial meningioma based on preoperative imaging, such as MRI or CT, and histopathological examination, to have undergone surgical resection performed at our institution, to be aged 18 years or older. Patients were excluded if they presented with multiple meningiomas or other concurrent intracranial tumors, had a history of prior cranial surgery or radiotherapy affecting the tentorium cerebelli, or suffered from severe comorbidities that could substantially influence perioperative management or outcomes, such as uncontrolled hypertension or advanced liver or kidney dysfunction. Furthermore, patients with incomplete or missing critical data (including daily postoperative pain scores and total analgesic consumption) in their medical records were also excluded. All data were extracted from the Hospital Information System (HIS) of the Second Affiliated Hospital of Zhejiang University.

Patients were divided into two groups: the control group and the CBZ group. In the control group, patients did not receive CBZ and were managed with analgesics as needed for pain relief. In the CBZ group, patients were treated with CBZ at a dosage of 200 mg twice daily after surgery, in addition to receiving analgesics as necessary for pain management.

### Perioperative analgesic protocol

All patients underwent standardized general anesthesia. Induction was achieved using intravenous propofol, sufentanil, and rocuronium, and anesthesia was maintained with sufentanil. Local anesthetic infiltration of the scalp incision was routinely performed at the end of surgery using 0.5% ropivacaine before wound closure. Postoperatively, all patients received a standardized analgesic regimen. Intravenous NSAIDs (such as flurbiprofen axetil 50 mg) were administered as first-line analgesics. Opioids (such as tramadol 50–100 mg intravenously) were provided as rescue analgesics when the Visual Analogue Scale (VAS) score exceeded 4. Analgesics were administered on demand based on patient-reported pain intensity rather than according to a fixed schedule.

### Data collection and outcome measures

The collected data were organized into several categories, including baseline demographic information, medical history, treatment-related variables, and complications during hospitalization. Baseline characteristics included age, sex, presence of hypertension or diabetes, length of hospital stay, postoperative C-reactive protein (CRP) levels, white blood cell (WBC) count and neutrophil percentage. In addition, we documented the total consumption of analgesics, including opioids and NSAIDs, throughout the recovery period. Each administration of analgesics was counted as one instance, and the total number of analgesic administrations was aggregated for final analysis. The primary outcome was postoperative pain intensity, assessed daily using the VAS during hospitalization (17). Patient underwent blood tests prior to discharge, with liver dysfunction was defined as alanine aminotransferase (ALT) and aspartate aminotransferase (AST) levels being 1.5 to 2 times above the normal range (18).

### Surgical approach

All surgeries were performed under a microscope with the patient's position chosen according to the tumor's location to optimize the surgical approach. The choice of surgical approach was determined by the tumor's site: For tentorial meningiomas confined to the midline and protruding into the posterior fossa, resection is performed via the midline supracerebellar infratentorial approach. Tumors located in the anterior region of the cerebellar tentorium are removed through the subtemporal approach. Tumors located near the transverse-sigmoid sinus can be addressed using the retrosigmoid approach. For tumors extending across both the supratentorial and infratentorial compartments, resection can be achieved via the occipital transtentorial approach or a combined supra- and infratentorial approach (19).

### Statistical analysis

Descriptive statistics were used to summarize the characteristics of the study population. Categorical variables were presented as frequency (percentage)., while continuous variables were expressed as mean ± standard deviation, depending on the distribution of the data. The independent t-tests were used to compare continuous quantitative variables between the groups, while the Chi-squares were used for categorical variables. All statistical analyses were conducted using IBM SPSS Statistics for Windows version 26.

## Results

A total of 42 patients were included in this study. The most common presenting symptoms were headache (16 cases) and dizziness (14 cases). Additionally, 8 patients were diagnosed with tentorial meningioma incidentally during routine physical examinations, while 3 patients exhibited gait instability, and 1 patient presented with hearing loss. The mean age of the enrolled patients was 53.1 years, with 31 women. There were no significant differences between the two groups in terms of hypertension, diabetes, postoperative inflammatory markers (including CRP, WBC, and neutrophil percentage). No significant differences were observed in liver function between the two groups prior to discharge. Additionally, the length of hospital stay in the CBZ group was shorter compared to the control group (10.45 ± 2.91 days vs. 10.55 ± 3.76 days, *p* = 0.145), and the total hospitalization costs were also lower (41,406.34 ± 7,912.66 RMB vs. 44,061.11 ± 16,767.16 RMB, *p* = 0.116). However, these differences did not reach statistical significance (Table 1).

**Table 1:** Baseline characteristics of patients with tentorial meningiomas.

Variables	Total (*n* = 42)	Control Group (*n* = 22)	CBZ Group (*n* = 20)	*p*-values
Age	53.12 ± 11.10	55.23 ± 11.33	50.80 ± 10.65	0.955
Female, *n* (%)	31 (73.8%)	17 (77.27%)	14 (70.00%)	0.592
Hypertension, *n* (%)	9 (21.43%)	6 (27.27%)	3 (15.00%)	0.333
Diabetes, *n* (%)	4 (9.52%)	2 (9.09%)	2 (10.00%)	0.920
Hospital Stay	10.50 ± 3.34	10.55 ± 3.76	10.45 ± 2.91	0.145
Postoperative CRP	32.18 ± 27.36	31.81 ± 28.72	32.62 ± 26.55	0.520
Postoperative WBC	13.90 ± 4.43	13.41 ± 4.29	14.45 ± 4.62	0.992
Postoperative *N*%	87.38 ± 5.58	87.11 ± 5.35	87.68 ± 5.94	0.625
Abnormal Liver Function before Discharge	8 (19.05%)	3 (13.64%)	5 (25.00%)	0.445
Total Hospital Charges	42,796.93 ± 13,221.68	44,061.11 ± 16,767.16	41,406.34 ± 7,912.66	0.116

Data presented as mean ± standard deviation or n (%). WBC, white blood cell; CRP, c-reactive protein; N%, neutrophilic granulocyte percentage. Total Hospital Charges were settled in RMB. The *p*-values were calculated using the independent samples t-test for continuous variables and the chi-square test for categorical variables.

**Figure 1:**
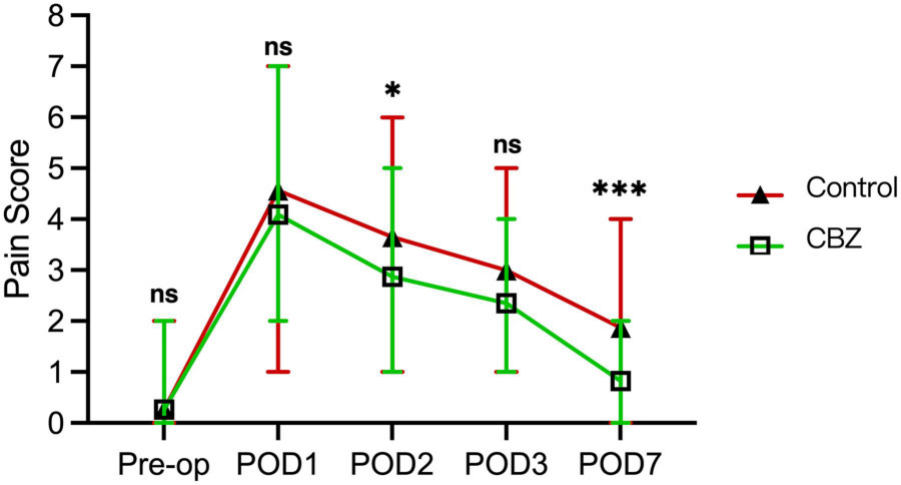
Comparison of perioperative pain scores between the CBZ group and the control group. The graph displays pain scores (scale 0-8) for both groups at different time points: Pre-op, POD1, POD2, POD3, and POD7. The CBZ group showed significantly lower pain scores compared to the control group on POD2 (*p < 0.05) and POD 7 (***p < 0.001). “ns” indicates non-significant differences.

Figure 1 illustrates the comparison of perioperative pain scores between the CBZ group and the control group. The preoperative VAS score did not differ significantly between the control and CBZ groups (*p* > 0.05). The pain scores in the CBZ group were significantly lower than those in the control group on both postoperative day 2 and day 7 (*p* < 0.05). This difference highlights the potential analgesic benefits of CBZ in managing postoperative pain. Specifically, the CBZ group demonstrated a more rapid reduction in pain intensity during the early postoperative period and maintained lower pain levels throughout the first week after surgery. These findings suggest that CBZ effectively alleviates pain, possibly by targeting the neuropathic component associated with tentorial meningioma surgery.

We analyzed the consumption of analgesic use within the first 96 h postoperatively and from 96 h post-surgery to discharge. Overall, the CBZ group required fewer analgesics compared to the control group (Table 2). During the first 96 h postoperatively, there was no significant difference between the two groups in the frequency of analgesic use (*p* = 0.212). However, from 96 h post-surgery to discharge, the CBZ group required significantly fewer analgesics than the control group (*p* = 0.043). Specifically, the use of opioids was significantly lower in the CBZ group (*p* = 0.023), while the difference in NSAIDs usage was not statistically significant (*p* = 0.228). The proportions of patients receiving combined NSAID and opioid therapy before discharge were comparable between groups (68.2% vs. 70.0%, *p* = 0.899), suggesting a balanced baseline analgesic strategy.

**Table 2:** Analgesic consumption between the control and CBZ group after surgery.

Variables	Total (*n* = 42)	Control Group (*n* = 22)	CBZ Group (*n* = 20)	*p*-values
Analgesic Used within 96 Hours After Operation	9.26 ± 2.82	9.50 ± 3.19	9.00 ± 2.41	0.212
Opioid Used within 96 Hours after Operation	2.86 ± 3.07	3.23 ± 3.28	2.45 ± 2.86	0.099
NSAIDs Used within 96 Hours after Operation	6.40 ± 3.49	6.27 ± 3.99	6.55 ± 2.95	0.110
Analgesic Used from 96 h to Discharge after Surgery	6.50 ± 4.16	7.05 ± 4.78	5.90 ± 3.37	0.043*
Opioid Used from 96 h to Discharge after Surgery	1.31 ± 2.68	1.73 ± 3.28	0.85 ± 1.79	0.023*
NSAIDs Used from 96 h to Discharge after Surgery	5.00 ± 3.77	5.14 ± 4.29	4.85 ± 3.22	0.228

Data presented as mean ± standard deviation. The *p*-values were calculated using the independent samples t-test for continuous variables. *p < 0.05.

## Discussion

Our study explored the role of CBZ in alleviating postoperative pain in patients with tentorial meningiomas. The results demonstrate that CBZ significantly reduces postoperative pain intensity, decreases the consumption of analgesics, and shortens the length of hospital stay. These findings suggest that CBZ offers an effective and safe adjunctive option for pain management in the perioperative period for this specific patient population. Effective pain control not only improves patient comfort but also contributes to better recovery outcomes by minimizing the potential complications associated with inadequate pain management, such as delayed mobilization and prolonged hospitalization. Importantly, our results showed no significant adverse effects associated with CBZ use, including liver function impairment, reinforcing its safety profile and suitability for use in the postoperative setting.

In the context of postoperative pain following neurosurgery, including tentorial meningioma resections, the pain experienced by patients often has a neuropathic component due to nerve irritation or injury during surgery (20). The efficacy of CBZ in managing postoperative pain is likely related to its well-documented neuro-modulatory properties. As a sodium channel blocker, CBZ stabilizes hyperexcitable neuronal membranes and modulates abnormal pain signaling (21), which may be particularly beneficial in cases of tentorial meningiomas. The tentorium cerebelli is innervated by branches of the trigeminal nerve (7), and surgery in this area can result in nerve irritation or inflammation, leading to postoperative pain that resembles trigeminal neuralgia. Conventional analgesics, such as NSAIDs and opioids, may not fully address this neuropathic component of the pain, whereas CBZ directly targets the underlying neural mechanisms. By modulating sodium channel activity, CBZ could reduce hyperexcitability in the nervous system, which makes it as a valuable addition to traditional pain management strategies, particularly for complex cases involving neuropathic pain (22, 23).

Our findings also highlight the potential benefits of incorporating CBZ into multimodal pain management protocols. Contemporary post-craniotomy pain management has increasingly evolved within Enhanced Recovery After Surgery (ERAS) frameworks, which emphasize multimodal analgesia, regional techniques such as scalp blocks, and opioid-sparing pharmacological strategies. As summarized in a comprehensive review of cranial ERAS pathways by Stumpo et al. (24), structured perioperative protocols integrating nonopioid analgesia and optimized anesthetic management are associated with improved postoperative pain control and reduced analgesic consumption. In this context, by reducing reliance on opioids and NSAIDs, CBZ may further contribute to help mitigate medication-related risks, including opioid dependence and NSAID-associated gastrointestinal or renal complications (25). These advantages underscore the importance of exploring mechanism-targeted interventions such as CBZ to optimize perioperative care in neurosurgical patients.

Postoperative pain management is essential for enhancing patients' quality of life and psychological well-being. Patients undergoing tentorial meningioma surgery may experience various types of pain, including surgical incision pain, headaches, and neuropathic pain (2). As part of a multimodal strategy, CBZ may be used alongside non-pharmacological treatments such as physical therapy and acupuncture to achieve more comprehensive pain control. Due to individual differences in drug response and tolerance to side effects, the use of CBZ requires personalized adjustment (26). Physicians will determine the appropriate dosage based on the patient's specific condition and closely monitor potential side effects, including drowsiness, dizziness, and tinnitus. However, no significant complications were observed in our study. While our findings suggest that CBZ may represent a valuable adjunct within ERAS-aligned multimodal strategies, further prospective studies are warranted to confirm its long-term efficacy and safety in postoperative pain management for tentorial meningioma.

However, this study has several limitations. As a single-center retrospective analysis, the findings may not be generalizable to broader populations, and causality cannot be firmly established. Selection bias cannot be entirely excluded. In addition, variability in surgical techniques among different surgeons may have influenced postoperative pain outcomes. Chronic headache disorders and pre-existing use of neuroleptic medications were not systematically excluded, which may have affected baseline pain perception and postoperative analgesic requirements. Although preoperative VAS scores were comparable between groups, residual confounding cannot be ruled out. Furthermore, despite similar baseline analgesic regimens, the retrospective design does not completely eliminate potential analgesic selection bias. Prospective, multicenter randomized studies with stricter eligibility criteria are needed to confirm these findings.

## Conclusion

In conclusion, this study suggests that CBZ is a safe and effective adjunct in managing postoperative pain in patients with tentorial meningiomas. Its ability to reduce analgesic requirements without significant side effects underscores its potential value in perioperative care. However, further research is needed to confirm these findings and establish standardized protocols for its use.

## Data Availability

The original contributions presented in the study are included in the article/Supplementary Material, further inquiries can be directed to the corresponding author.
